# Balance Between Order and Disorder: Quantum-Epigenetic Pathways for the Emergence and Continuous Evolution of Life

**DOI:** 10.1007/s10441-026-09541-8

**Published:** 2026-09-16

**Authors:** Matheus Correia Casotti, Camilly Victoria Campanharo, Daniel Moreira Melo, Debora Gonçalves Barbosa, Gabriel da Cruz Simões, Isabele Pagani Pavan, Lorena Souza Castro Altoé, Lorena Souza Rittberg Mauricio, Maria Eliza Soares Queiroz, Tamires de Almeida Angelos, Victor Alves Lopes, Yasmin Moreto Guaitolini, Adriana Madeira Álvares da Silva, Cleocir José Dalmaschio, Marcelo Victor Pires de Sousa, Rafael de Queiroz Ferreira, Flavia de Paula, Iúri Drumond Louro, Débora Dummer Meira

**Affiliations:** 1 Universidade Federal do Espírito Santo (Ufes), Vitória, ES Brazil; 2 Instituto Federal de Sergipe (IFS), Campus Estância, Estância, SE Brazil; 3 Bright Photomedicine, Tergos Pesquisa & Ensino S.A, São Paulo, SP Brazil; 4 Instituto D’Or de Pesquisa e Ensino – Ciência Pioneira, Rio de Janeiro, RJ Brazil

**Keywords:** Life, Epigenetics, Ploidy variation, Cell fate, Biotechnology, Astrobiology

## Abstract

The scientific restlessness surrounding the question of what life is (expressed in the provocations of Feynman and Schrödinger) guides this review article by integrating physics, chemistry, and biology into a multidimensional and emergent approach to the origin, evolution, and recreation of life. By exploring classical and quantum concepts, we discuss how synthetic biology, combined with epigenetics, ploidy variation, and quantum effects (such as coherence and tunneling) offers new possibilities for adapting and designing living systems. This review covers a spectrum of topics, from prebiotic chemistry experiments and the construction of protocells to practical applications in regenerative medicine, bioengineering, and astrobiology. It highlights how changes in the number of chromosome sets influence cellular plasticity and shape dynamic epigenetic environments, fostering the emergence of adaptive properties. It also explores how such factors could steer regeneration, aging, cancer development, and the expansion of life into extreme and extraplanetary environments. Based on an integrative review of scientific literature, relevant publications were selected on topics such as biological complexity, molecular organization, and adaptive strategies, with emphasis on the convergence between natural and artificial life. By considering the balance between disorder and order as a driving force of evolution, this review proposes that a deep understanding of life, anchored in a quantum-epigenetic perspective, is essential for its manipulation, restoration, and expansion. Thus, it suggests that the key to designing new forms of life lies in understanding complexity as the foundation for biotechnological innovation.

## Introduction

Over the years, the quest to understand life-whether to create it, evolve it, or even restore it-has shaped generations of scientific research and inspired works of science fiction. Scientists such as Richard Feynman, who declared, “What I cannot create, I do not understand,” and Erwin Schrödinger, with his famous question, “What is life?”, sparked profound debates about the nature and limits of life (Olby 1971). These inquiries lead us to an even more complex reflection: could the integration of concepts from physics, chemistry, and biology-by merging classical and quantum paradigms-ignite a new scientific revolution? In the contemporary landscape, the emerging frontiers of synthetic and quantum biology within biotechnology suggest new paths for understanding life, its origins, and its ongoing processes of repair and adaptation (Lombardo 2005; Longo et al. 2012).

Despite remarkable advances in defining and investigating life-from the classic experiments of Miller and Oro that demonstrated the synthesis of amino acids and nucleotides under prebiotic conditions (Huang 2004; Solé 2016) to current synthetic biology creations of protocells-gaps persist in conventional approaches. These include understanding how changes in the number of chromosome sets (“ploidy variation”) can trigger deep epigenetic reprogramming, generating dynamic genomic microenvironments (Sanchez and Mackenzie 2023). Such changes promote morphofunctional adaptations, even at subatomic levels-such as the induction of tautomeric states in DNA-potentially linked to the generation of point mutations (Löwdin 1963).

Amidst these reconfigurations, despite the presence of chaos and disorder, there remains a biological coherence that promotes order from order, as emphasized by Robert (2013). Thus, emergent properties arise, opening possibilities to explore multiple “levels of organization,” maximizing resilience and adaptive innovation, such as through the use of interconnected dimensions of ploidy, epigenetics, and quantum phenomena, as highlighted by Bedau (2003) and Pezzulo and Levin (2016). In this context, technological developments are advancing, enabling the development of artificial biological systems through bottom-up, top-down, and middle-out approaches-making in situ resource production and even the colonization of exoplanets feasible (Rothschild et al. 2024). By understanding complexity, it becomes possible to control it and even drive the investigation of new possibilities for designing life.

Based on the aforementioned considerations, this article aims to integrate concepts from chemistry, physics, and biology to explore a central question: Is it possible to adapt the life we know into new forms of life through epigenetic and quantum balancing, driven by organismal evolution stemming from ploidy variation? To address this question, the Results and Discussion section of this review is organized into six thematic subsections that progressively build an integrative narrative. The first subsection discusses the fundamental concepts of life, covering thermodynamic, functional, and emergent definitions, as well as the historical and theoretical roots of these ideas. The second subsection explores the role of ploidy, including its prokaryotic origins, and cellular plasticity in maintaining tissue homeostasis and enabling phenotypic transitions. The third subsection focuses on the relationship between epigenetic mechanisms, genetic instability, and cell fate determination, with ploidy variation functioning as an integrative link. The fourth subsection deepens this discussion by examining potential quantum-level implications for cellular decision-making, culminating in the proposal of the Quantum-Epigenetic Field (QEF) hypothesis. The fifth subsection extends these concepts to extreme contexts, such as tissue regeneration, artificial life engineering, and astrobiology. Finally, the sixth subsection synthesizes these threads into an integrative model of “life in the quantum era,” highlighting the challenges and opportunities for exploring and (re)designing life forms on Earth and beyond.

## Methodology

An integrative review was conducted based on articles addressing the multidimensional aspects of life. The search strategy employed encompassed philosophical, biological, chemical, physical, and technological perspectives, and was conducted using the following query: (((“definition of life” OR “philosophical biology” OR “biological existence” OR “emerging criteria”) AND (“biological complexity” OR “molecular organization” OR “cellular organization” OR “systemic organization” OR “evolutionary processes” OR “emergence”)) AND ((“stress biology” OR “regeneration” OR “recovery” OR “aging” OR “senescence” OR “complex diseases” OR “cancer” OR “resilience”) AND (“adaptive systems” OR “artificial life” OR “synthetic biology” OR “bioengineering” OR “evolutionary strategies”)) AND ((“biological limits” OR “biochemical barriers” OR “energetic constraints” OR “structural constraints” OR “extreme life”) OR (“extreme environments” OR “extremophiles” OR “astrobiology” OR “biosignatures” OR “exoplanets” OR “interplanetary adaptation”)) AND ((“technological advancements” OR “innovations in biology” OR “convergence of natural and artificial life” OR “future paradigms”))). To make this Boolean structure more transparent, the query can be summarized as the conjunction of seven thematic blocks, detailed in Table 1: (Topic 1: definitions and criteria of life) AND (Topic 2: principles of biological, molecular, and systemic organization and evolutionary processes) AND (Topic 3: pathological and adaptive contexts, such as regeneration, aging, and cancer) AND (Topic 4: adaptive, artificial, and synthetic biological systems) AND [(Topic 5: biophysical, biochemical, and energetic limits of life) OR (Topic 6: extreme environments and astrobiology)] AND (Topic 7: technological convergence and future paradigms). Because Topics 1–4 and 7 are combined with the AND operator while the alternative terms within each topic are combined with OR, an individual retrieved article was not expected to simultaneously address every topic (e.g., cancer and extremophiles) in depth; rather, each article needed only to intersect with at least one term from each block, and the resulting corpus was subsequently curated for thematic coherence during full-text screening. The database used for this search was Google Scholar, and exclusion criteria included book chapters, dissertations, theses, and full books. Original research articles, review articles, conference proceedings, and preprints were included. Titles and abstracts were evaluated based on thematic relevance in line with the research objectives, with an emphasis on an integrated bibliography. In addition to the automated search, manual inclusion of relevant publications was also performed. The selected full-text articles were analyzed and synthesized to construct a comprehensive and coherent narrative on the themes of this review. This approach ensured a broad yet focused perspective, providing a solid foundation for discussing the complexity of life and its potential biotechnological applications. Not every one of the 112 selected articles is individually cited in each subsection of the Results and Discussion; rather, they constitute the curated corpus from which the arguments developed throughout this review were drawn. This corpus was complemented, where explicitly indicated, by a small number of additional foundational works identified through the manual inclusion process described above, used to address specific conceptual gaps (e.g., the origin of life, prokaryotic ploidy, eukaryogenesis, and developmental bioelectricity) that were not fully captured by the original search terms.

**Table 1:** Thematic decomposition of the Boolean search query used in this integrative review

Topic	Description and example search terms	Role in Boolean query
1	Definitions and criteria of life - “definition of life”, “philosophical biology”, “biological existence”, “emerging criteria”	AND Topic 2
2	Biological, molecular, and systemic organization - “biological complexity”, “molecular organization”, “cellular organization”, “systemic organization”, “evolutionary processes”, “emergence”	AND Topic 1
3	Pathological and adaptive contexts - “stress biology”, “regeneration”, “recovery”, “aging”, “senescence”, “complex diseases”, “cancer”, “resilience”	AND Topic 4
4	Adaptive, artificial, and synthetic biological systems - “adaptive systems”, “artificial life”, “synthetic biology”, “bioengineering”, “evolutionary strategies”	AND Topic 3
5	Biophysical, biochemical, and energetic limits of life - “biological limits”, “biochemical barriers”, “energetic constraints”, “structural constraints”, “extreme life”	OR Topic 6 (block then AND with Topics 1–4 and 7)
6	Extreme environments and astrobiology - “extreme environments”, “extremophiles”, “astrobiology”, “biosignatures”, “exoplanets”, “interplanetary adaptation”	OR Topic 5
7	Technological convergence and future paradigms - “technological advancements”, “innovations in biology”, “convergence of natural and artificial life”, “future paradigms”	AND with the whole conjunction

## Results and Discussion

Based on the literature search conducted through Google Scholar, 250 articles were identified, of which 112 were selected according to the inclusion and exclusion criteria, as well as their relevance to the subject matter and objectives of this review. Focusing on the integration of concepts such as ploidy variation, cell fate, biological regeneration, senescence, aging, rejuvenation, and chronic diseases-particularly cancer-the results were organized to construct a progressive conceptual narrative. The discussion begins with definitions and foundational principles of the concept of life. Next, ploidy variation is presented as an integrative link between different biological processes, proposed as a potential theoretical pillar for future studies on the understanding of life. The discussion then explores how the environment influences cellular and organismal adaptation in response to ploidy variation. Finally, a multiscale analysis is proposed-from the quantum to the macroscopic level-on how life has adapted, and can continue to adapt, to different environments, considering ploidy variation as a bridge between distinct “worlds”: the classical (Newtonian), thermodynamic, and quantum realms.

1) Definitions and Concepts of Life.

Life is often defined as a self-sustaining chemical system capable of exchanging energy and information with the environment and evolving through natural selection (Des Marais et al. 2008). This functional definition, adopted by institutions such as NASA (National Aeronautics and Space Administration), highlights the importance of nonequilibrium states, in which energy dissipation and physicochemical interactions drive organization, adaptation, and complexity. Other widely used criteria for characterizing life include compartmentalization, metabolism, and replication. However, the existence of cryptobiotic organisms and structures like dormant seeds challenges these traditional categories, pointing to the need for a more pluralistic and flexible approach (Helman 2022; Sánchez-Cañizares 2016; Gómez-Márquez 2023).

Long before Schrödinger posed his famous question, naturalists and embryologists had already grappled with how form and order arise in living systems. In the early twentieth century, Hans Driesch’s experiments on sea-urchin embryos and the discovery, by Hans Spemann and Hilde Mangold, of an embryonic “organizer” capable of redirecting the developmental fate of surrounding tissue suggested that morphogenesis is governed by field-like, non-local influences rather than by a simple, linear unfolding of a molecular blueprint-ideas that were subsequently articulated within the organicist embryological tradition as the concept of the morphogenetic field, and that have more recently been reformulated in terms of bioelectric pattern control (Levin 2021). These early organicist traditions anticipated, in a pre-molecular vocabulary, the systemic and field-based perspectives that later authors would attempt to ground first in thermodynamics and, more recently, in quantum theory, as discussed throughout this review.

Building on these systemic traditions, several proposals have sought to refine or extend the classical, largely molecular, definitions of life mentioned above. Assembly theory, for instance, characterizes objects by their “assembly index” (the minimum number of recursive steps required to build a complex molecule from simpler precursors) proposing that the abundance of high-assembly-index molecules can serve as a measurable, substrate-independent signature of selection and, by extension, of life, without presupposing terrestrial biochemistry (Sharma et al. 2023). Likewise, the dissipative-adaptation framework proposes that, under a persistent external energy source, configurations of matter that absorb and dissipate more work tend to be dynamically favored over time, providing a physical rationale for why complex, life-like structures might emerge spontaneously in driven, non-equilibrium systems (England 2013). Together with the thermodynamic and autopoietic criteria mentioned above, these newer frameworks reinforce a view of life centered on self-organization, dissipation, and informational complexity rather than on any single, fixed molecular inventory.

Authors such as Skene (2024) and Simons (2021) argue that the organization of life is deeply rooted in thermodynamic principles, particularly in energy efficiency and entropic processes that shape life forms from the simplest to the most complex multicellular organisms. These principles directly influence the molecular structure and evolution of proteins, reflected in the operation of the genetic code and the self-organizing mechanisms of biological systems. Comparable thermodynamic readings of life, which treat order, complexity, and organization as consequences of energy flow through open systems rather than as properties added to matter, have been developed in parallel by Egel (2012) and Palmer (2023).

These self-organization and functional delimitation processes are associated with the notion of autopoiesis, whereby biological systems maintain their integrity through the formation of physical boundaries and internal regulation (Sánchez-Cañizares 2016; Helman 2022). Models such as chiral amnesia and crystal engineering illustrate plausible prebiotic mechanisms for enantiomeric enrichment, contributing to the molecular organization necessary for the emergence of life with Darwinian capacity (Sánchez-Cañizares 2016; Helman 2022). In this context, homochirality functions as an anchor point between molecular structure and biological functionality.

From a systemic perspective, life can be understood as an emergent and multiscale phenomenon resulting from the integration of dissipative structures and dynamic interaction networks. According to the ideas of Prigogine and Dodig‑Crnkovic (2024), the complexity of living systems emerges from self-organized mechanisms operating from the molecular level up to the formation of tissues and organisms. This view is complemented by Saetzler et al. (2011), who advocate for moving beyond reductionist approaches and propose an analysis capable of capturing the bidirectional interactions and collective behaviors that characterize living beings. In the same vein, Brown and Hullender (2023) argue that biological evolution is unintelligible without an emergent, self-organizing principle, while Wegner (2024) proposes a formal vocabulary of systems, emergence, and metafluxes for describing such multiscale organization.

Additionally, historical experiments such as those by Stanley Miller and Joan Oro in 1953 demonstrated that amino acids and nucleotides can arise under simulated prebiotic Earth conditions. In other words, autocatalytic networks and dissipative structures also contribute to our understanding of life (Huang 2004; Solé 2016). The informational dimension of these prebiotic transitions-how molecular assemblies come to carry, preserve, and transmit information-remains a central open question in this literature (Chirumbolo and Vella 2021). With technological advances, synthetic biology expands these possibilities by introducing new approaches to creating artificial life, such as: bottom-up (building life from scratch); top-down (simplifying existing organisms); and middle-out (combining functional modules) (Ellis et al. 2012; Longo et al. 2012; Ellis 2018; Göpfrich et al. 2018).

Other models suggest that the origin of life may have occurred in highly dispersed colloidal masses subject to environmental entropic variability, where inorganic and organic molecules interacted with various energy sources. This combination of factors could have favored the emergence of dissipative networks and the formation of ordered structures, mediated by mechanisms of quantum coherence and decoherence, as proposed by Spitzer (2024) and Matsuno and Nemoto (2005). From this perspective, quantum dynamics may have played an essential role by allowing exchanges between ordered and disordered states, thus favoring the emergence of self-organized structures with functional capacities.

A substantial body of work situates the origin of life itself within this dissipative, self-organizing framework, with particular emphasis on the role of naturally occurring energy gradients. The alkaline hydrothermal vent theory, developed principally by Russell, Martin, and Lane, proposes that steep pH and redox gradients across thin, inorganic Fe(Ni)S membranes-formed where alkaline hydrothermal fluids mix with the more acidic Hadean ocean-could have driven the earliest carbon- and energy-fixing reactions, providing a geochemical template for the proton-motive, chemiosmotic machinery that powers virtually all extant cells (Martin and Russell 2007; Martin et al. 2008; Harrison et al. 2022). In this view, life did not need to invent the proton gradient from scratch; rather, it internalized and progressively refined a pre-existing geophysical disequilibrium through increasingly self-contained autocatalytic networks (Martin and Russell 2007). More generally, if the emergence of life is understood as an autocatalytic, dissipative process, then increasing biological complexity can be interpreted as a means of accelerating the dissipation of available energetic potentials-whether geothermal, chemical, or solar-consistent with broader proposals that self-organized, far-from-equilibrium systems tend to evolve toward configurations that enhance entropy production (Skene 2024). Early formal treatments of self-organization in complex systems, such as Langton’s (1990) analysis of computation at the “edge of chaos,” remain influential in framing how systems poised between order and disorder can support the kind of adaptive information processing discussed throughout this review. Under this framing, life would not be confined to Earth-like conditions but would be expected to emerge wherever comparable disequilibria and dissipative pathways exist, with its specific molecular forms canalized by the physicochemical constraints of each environment-a point we revisit in the context of astrobiology below. The passage from complex prebiotic chemistry to genuinely biological organization has been analysed in detail by Ruiz-Mirazo and Moreno (2023), while Solé et al. (2024) argue that the space of possible living systems is bounded by a small set of fundamental physical and computational constraints, which limits how far this diversity can extend.

In alignment with previous perspectives, cooperation is another fundamental principle that transcends the various scales of life. The theory of symbiogenesis, proposed by Lynn Margulis, emphasizes symbiotic fusion as a driving force in cellular evolution, particularly in the origin of eukaryotic cells (Goldenfeld and Woese 2011). This perspective is supported by experiments with amoebas and fungi, which demonstrate the role of cooperative associations in the emergence of multicellularity (Sagan 2021). Modern approaches in synthetic biology draw inspiration from these natural processes to develop artificial microsymbionts aimed at creating life forms based on the integration of diverse biological components.

The synergy between distinct parts is also central to biological complexity. The CMP model (Container, Metabolism, and Genetic Program), proposed by Corning (2022), maintains that the coordinated integration of physical compartments, metabolic flows, and genetic information systems was essential for the evolution of multicellular organisms. Examples such as cooperative hunting by Homo erectus illustrate how collaboration among individuals has shaped both biological evolution and human cultural systems (Corning 2022).

Given the many definitions and dimensions of life and its origin, a collaborative and transdisciplinary approach proves essential. This could enable advances in various fields, such as the use of symbiotic enzymes and microorganisms adapted to extreme environments to facilitate the colonization of exoplanets-both through in situ resource production and the regeneration of living systems in “alien” contexts (Kauffman 1995; Harrison et al. 2022). Thus, recognizing convergent patterns that situate life as an emergent, organized, resilient, and cooperative process-constantly adapting to the energetic and environmental conditions that sustain it-is crucial. Furthermore, elucidating the mechanisms that contribute to life’s resilient balance, such as ploidy variation, may lead to new mechanistic understandings.

2) Ploidy as a mechanism of biological flexibility.

Immersed in what can be defined as life, ploidy-understood as the number of complete sets of chromosomes in a cell-represents a fundamental mechanism of biological flexibility. Alterations in this number directly influence cellular function and fate, as well as the adaptability of organisms to variable environments (Casotti et al. 2024, 2025). These variations, often treated as exceptions, are in fact central to the biology of regenerative tissues, cancer, and various evolutionary processes (Casotti et al. 2024, 2025).

Although ploidy variation is often discussed in the context of eukaryotic tissues, the underlying logic of genome copy-number flexibility long predates the emergence of eukaryotes. For roughly two billion years, life on Earth existed exclusively as prokaryotes, and many bacteria and archaea are themselves polyploid or maintain multiple chromosome copies per cell-examples include the radiation-resistant bacterium Deinococcus radiodurans and several cyanobacteria, in which genome copy number varies with growth phase and environmental stress (Anatskaya and Vinogradov 2022). Cooperative, differentiation-like behavior also predates eukaryotes: clonal populations of bacteria and archaea can display spatial and temporal patterns of gene expression that generate phenotypically distinct subpopulations within a single colony, foreshadowing the division of labor seen in modern multicellular tissues (Engelberg-Kulka et al. 2006); the evolutionary origin of distinct cell types from such ancestral regulatory repertoires has been reconstructed in detail by Arendt et al. (2016). Programmed cell death, too, has prokaryotic roots-toxin–antitoxin modules, such as the mazEF system of Escherichia coli, allow a fraction of cells in a stressed population to die so that the remainder survive, an altruistic, population-level strategy that foreshadows the apoptotic programs of multicellular organisms (Engelberg-Kulka et al. 2006). The transition to the eukaryotic cell itself appears to have emerged from a metabolic and symbiotic partnership between an archaeal host and a bacterial partner, as proposed in the hydrogen hypothesis and supported by the discovery of Asgard archaea such as Lokiarchaeota, which possess many of the molecular precursors of eukaryotic cellular complexity (Martin and Müller 1998; Spang et al. 2015). From this standpoint, the ploidy variation and associated epigenetic and bioenergetic consequences discussed throughout this review are not late evolutionary inventions layered onto an already-complex eukaryotic cell, but rather extensions of regulatory strategies for managing genomic dosage, cooperation, and cell fate that were already present, in simpler form, among the prokaryotic ancestors of all extant life.

In nature, genomic plasticity is observed from unicellular organisms to complex systems. Arthropods that undergo complete metamorphosis illustrate how discontinuous evolutionary patterns-such as genomic reorganizations and ploidy modulation-can sustain morphofunctional innovations (Igamberdiev and Gordon 2023). In this context, Gregory and Hebert (1999) and Sharov (2006) argue that processes such as polyploidization are associated with discontinuous evolutionary leaps. Such transitions reflect abrupt shifts between stable genomic states, which can be modeled as transitions between epigenetic attractors (Feinberg and Levchenko 2023). These changes are connected to the nonlinear dynamics of evolutionary biology, consistent with a view of evolution in leaps, combining micro- and macro-adaptive transitions (Igamberdiev and Gordon 2023). Comparative work on chromosome fusions in plants provides a concrete karyotypic record of such discontinuous reorganizations (Lysak 2022), and the theoretical status of these saltational mechanisms within evolutionary theory remains actively debated (Bojinov 2024).

In pathological contexts such as cancer, ploidy plays a critical role. Previous reviews by various authors-including Alves et al. (2023), Meira et al. (2023), Casotti et al. (2023, 2024, 2025), and Uchiya et al. (2025)-have highlighted that polyploid tumor cells can form syncytia and other multinucleated/mononucleated structures in different tumor types, with numerous impacts, reinforcing cellular plasticity and resistance to treatments. This adaptability is supported by deep epigenetic reprogramming-including DNA methylation and histone modifications-whose instability can be interpreted as a form of electronic decoherence (Gustin et al. 2023). Thus, genomic instability ceases to be merely a marker of dysfunction and becomes understood as a driver of evolutionary and phenotypic flexibility. An alternative, and increasingly influential, framework for interpreting these adaptive phenotypes is the atavistic theory of cancer, which proposes that malignant transformation represents the reactivation of ancient, evolutionarily conserved survival and proliferation programs that predate multicellularity, rather than being solely the product of stochastically acquired mutations (Davies and Lineweaver 2011). Within this framework, the genomic and ploidy instability discussed above can be understood not merely as damage but as a partial return to a more “primitive,” polyploidy-tolerant cellular repertoire that was already present in the unicellular and early multicellular ancestors discussed above.

In other contexts, such as the relationship between metabolism, ploidy, and cell fate, this connection is particularly evident in regenerative tissues, such as the liver and hematopoietic cells. Hematopoietic and hepatic models show that stem cells remain quiescent under hypoxic conditions, sustained by glycolytic metabolism. During differentiation, these cells adopt an oxidative metabolism (OXPHOS)-a reprogramming also evident in the regenerating liver, where beta-oxidation and lipid biosynthesis dynamically adjust to the cell’s ploidy state (Zhang et al. 2019; Addissouky 2024; Yin et al. 2024). An analogous coupling between genome copy number and metabolic adaptation has been described in tumours, where polyploid and multinucleated giant cells remain metabolically active during dormancy and later repopulate the tumour (Mirzayans et al. 2018), and where extensive rewiring of energy metabolism accompanies malignant progression (Delgado-Martín and Medina 2020). In the regenerating liver, polyploid hepatocytes modulate their metabolic profiles based on their genomic state and can activate c-MYC, promoting nucleotide biosynthesis and boosting cellular energy. In a pathological setting, however, the same signalling axes-particularly receptor tyrosine kinase–Ras–PI3K–Akt signalling-can sustain epithelial–mesenchymal transition (EMT) and treatment resistance (Tuncel and Kalkan 2018; Fabro et al. 2022).

Beyond metabolic changes, the cell cycle directly influences cell fate. In pluripotent cells, the length and regulatory state of the G1 phase modulate sensitivity to differentiation signals, so that an identical stimulus can produce different lineage outcomes depending on the point in the cycle at which it is received; this cell-cycle dependence of fate choice is one expression of the broader epigenetic plasticity that governs transitions between cellular states (Feinberg and Levchenko 2023). In hepatocytes, these dynamics can be modulated by complexes such as cyclin D–CDK4/6, directly influencing genomic stability and the propensity for differentiation or malignancy; deregulation of the same cell-cycle and growth-factor axes is a recurrent feature of tumour progression (Khabibov et al. 2022).

Additionally, computational and logic-based approaches have contributed to the mapping and control of these cellular processes. High-dimensional dynamic models based on differential equations and stochastic dynamics allow cellular states to be represented as attractors in epigenetic landscapes (Huang 2004; Dinicola et al. 2011; Feinberg and Levchenko 2023). Two distinct mechanisms must be distinguished at this point, since an attractor is strictly defined only for a fixed set of parameters and the deterministic dynamics alone cannot move a system out of a basin of attraction. Transitions therefore require either a change in the control parameters of the system-ploidy, chromatin state, or the metabolic and bioenergetic conditions of the cell-which deforms the landscape and can cause an attractor to lose stability or disappear through a bifurcation; or a stochastic fluctuation large enough to carry the trajectory across a separatrix into a neighbouring basin. In the second case the states are more properly described as metastable rather than as strictly asymptotic attractors, and the relevant quantity is the mean first-passage time between basins, which grows exponentially with the height of the quasi-potential barrier that separates them (Aon et al. 2011). Both mechanisms are pertinent to the argument developed here: ploidy variation and epigenetic remodelling act primarily as parameter changes that reshape the landscape, whereas transcriptional noise sets the escape rate from a basin once the landscape is fixed. Estimated transition times consequently provide a metric for the robustness or fragility of cellular states-a point of particular relevance in polyploid hepatocytes, whose regenerative or pathological responses depend on the stability of these states.

This computational perspective is reinforced by control-theoretic strategies applied to intracellular networks, which allow critical regulatory nodes to be identified and the biological system to be steered toward desired attractors (Pezzulo and Levin 2016; Feinberg and Levchenko 2023). Such interventions are particularly promising, enabling the reversal of senescence or promoting regeneration without permanent genomic modifications. This regulatory plasticity is notably reflected in hepatocytes, where different ploidy states offer multiple response pathways to external stimuli, providing strategic phenotypic flexibility in contexts of injury or chronic inflammation (Zhang et al. 2019; Yin et al. 2024).

The flexibility promoted by ploidy can also be understood in light of molecular emergence and self-organization processes. Studies such as those by Spitzer (2024) and De la Fuente et al. (2021) highlight the formation of proto-cellular structures based on coherence, thermal instability, and colloidal organization-fundamental elements for the emergence of self-regulatory behaviors and dissipative structures. Such systems, based on non-equilibrium dynamics, evolve toward complexity through interactions between molecules, energy flow, and primitive metabolic cycles. Experimental work on non-equilibrium self-assembly has since shown that purely chemical systems, when continuously supplied with fuel, can display transient, self-repairing, and adaptive behaviours that were previously regarded as diagnostic of living matter (Singh et al. 2024).

Together, Igamberdiev and Gordon (2023) emphasize that stable non-equilibrium states may enable the emergence of new levels of biological organization. For instance, the acquisition of mitochondria in eukaryotes marked a bioenergetic leap that enabled more complex cell divisions and greater adaptation to hostile environments (Goldenfeld and Woese 2011). In prokaryotes, mechanisms such as horizontal gene transfer and morphological variations sustain evolutionary innovation under different selective pressures (Goldenfeld and Woese 2011).

3) Influence of epigenetics on cell fate determination.

Epigenetics represents one of the main regulatory mechanisms of gene expression without altering the DNA sequence, operating through chemical modifications such as DNA methylation, histone acetylation, and changes in chromatin structure. These mechanisms function as contextual regulators that modulate access to genetic material and, consequently, determine cell identity (Jorgensen 2011; Feinberg and Levchenko 2023). In complex biological systems-such as regenerative tissues and tumors-epigenetics plays a central role in phenotypic plasticity, enabling cells to transition between different functional states in response to internal and external stimuli (Jorgensen 2011; Feinberg and Levchenko 2023).

In cancer, for example, the highly dynamic and stochastic epigenetic environment creates molecular landscapes characterized by biological noise and high transcriptional variability. Single-cell techniques, such as scRNA-seq (single-cell total RNA sequencing), show that this variability is intrinsic to cellular adaptation processes and can be interpreted as an increase in system entropy-an indicator of the expansion or emergence of new cellular attractors (Feinberg and Levchenko 2023). This plasticity not only favors tumor survival under selective pressure but also contributes to phenotypic heterogeneity and therapeutic resistance.

The interaction between ploidy and epigenetics adds an additional layer of complexity to the control of cell differentiation. This is because ploidy variation alters the structural organization of the genome and the DNA microenvironment, directly impacting epigenetic patterns (Jorgensen 2011; Feinberg and Levchenko 2023; Hatakeyama and Ohbayashi 2024). These alterations modulate not only gene expression but also influence quantum processes, such as proton tunneling and the formation of tautomeric states-as proposed by Löwdin (1963) and further explored by Slocombe et al. (2021). These dynamics suggest that ploidy does not act in isolation but interacts with epigenetic and physicochemical mechanisms that govern genome stability and cell fate.

This interplay is particularly evident in hepatocytes, in which ploidy variation is physiological and plays an adaptive role in regenerative processes. Polyploid hepatocytes arise through mechanisms such as endoreduplication, cytokinesis failure, or cell fusion, becoming predominant after birth or under hepatic stress conditions. Studies by Wilkinson et al. (2019) and Donne et al. (2021) show that hepatocyte ploidy is dynamically regulated during regeneration and chronic injury: diploid hepatocytes enter the cell cycle more rapidly, whereas the polyploid state acts as a brake on proliferation while conferring protection against genotoxic damage and malignant transformation, with ploidy reversal allowing the tissue to recover proliferative capacity when required. In both directions, these adjustments are implemented through coordinated epigenetic and metabolic changes that preserve the functional robustness of the liver. These cells modulate crucial pathways such as PI3K/AKT (Phosphatidylinositol 3-kinase/Protein kinase B-signaling pathway related to metabolism and cell growth), LKB1/AMPK (Liver kinase B1/AMP-activated protein kinase-a signaling pathway acting as a metabolic checkpoint), and Hippo-YAP (Hippo kinase/Yes-associated protein 1-a pathway involved in tissue development and homeostasis), promoting cell survival and plasticity under adverse conditions; the deregulation of these same axes is likewise central to survival and therapy resistance in tumours of other tissues (Khabibov et al. 2022; Fabro et al. 2022).

Other studies on polyploid cells, such as PGCCs (Polyploid Giant Cancer Cells), further deepen this relationship. Work by Lin et al. (2019), White-Gilbertson et al. (2020, 2022), and Mirzayans et al. (2018) reveals that these cells exhibit remarkable epigenetic and metabolic plasticity, sustaining highly adaptive behaviors such as therapy resistance and tumor-regenerating capacity. This logic also applies to hepatocytes, which adjust their functional plasticity based on genomic load through a coordinated response involving metabolism, epigenetics, and nuclear organization (Zhang et al. 2019).

It is also important to recognize that the control of cell fate described above cannot be reduced to genetic and epigenetic information alone. Work on developmental bioelectricity, particularly by Levin and colleagues, has shown that spatial patterns of transmembrane voltage and ion flux across groups of cells constitute an additional, physically grounded layer of information that instructs gene expression, tissue patterning, and even large-scale anatomical outcomes, often largely independently of any specific gene sequence (Levin 2021). From this perspective, bioelectric and morphogenetic fields-rooted in electromagnetic phenomena and ion/proton flux rather than in the genome per se-represent a complementary, and in some respects more fundamental, substrate of biological information than the gene-centric framework that has historically dominated molecular biology. Reframing ploidy-driven epigenetic reprogramming within this broader bioelectric context suggests that genomic dosage changes may act, at least in part, by altering the bioelectric properties of cells and tissues, with downstream consequences for gene expression that are mediated by physics before they are mediated by biochemistry-a perspective consistent with the Quantum-Epigenetic Field (QEF) framework developed later in this review.

From a physicochemical and thermodynamic perspective, epigenetics emerges as a dissipative system operating far from equilibrium, shaped by fluctuations and symmetry breaking (Simons 2021; Skene 2024). Sanchez and Mackenzie (2023) explore how DNA methylation patterns, mediated by DNMTs (DNA methyltransferases) and TETs (Ten-Eleven Translocation enzymes), are organized on mesoscopic scales to form an “epigenetic clock” that governs cell fate decisions. This approach emphasizes that epigenetic organization is not static but dynamically responds to structural genome variations, including those caused by changes in ploidy.

Moreover, enzymes play an active role in this epigenetic and metabolic regulation, functioning as nanomachines that catalyze chemical reactions far from equilibrium and enable the emergence of self-organized structures (De la Fuente et al. 2021). De la Fuente et al. (2021) describe enzymes as informational units that connect biological memory, metabolic coherence, and the transmission of cell states, shaping cyclical patterns of epigenetic inheritance and functional reorganization. This memory, according to Pontes (2016) and Katsnelson et al. (2018), can be modeled by Hopfield-type dissipative networks, in which emergent properties regulate cell plasticity and response to environmental stimuli.

In this framework, Asano et al.’s (2013) quantum-like theory proposes that epigenetic states can be formally represented as vectors in Hilbert spaces, subject to decoherence processes induced by the cellular environment. These superposed states evolve into statistical mixtures, and entanglement among epigenetic markers may accelerate “evolutionary leaps” through molecular synchrony, connecting Darwinian, Lamarckian, and Wrightian models within a unified structure. This perspective reinforces that epigenetic processes, influenced by ploidy, operate as open systems in constant reorganization, sensitive to quantum microstates and environmental conditions.

Ultimately, epigenetics provides the means by which the cell interprets, responds to, and reprograms its function in different contexts. The hepatic model, in particular, offers an ideal microcosm to study this intersection between ploidy and epigenetics, revealing how genetically identical cells can adopt diverse and adaptive functional profiles (Zhang et al. 2019; Anatskaya and Vinogradov 2022; Addissouky 2024; Aykan 2024; Yin et al. 2024). In this way, cellular plasticity is understood not as a deviation from the norm but as a regulated expression of biological complexity, sustained by epigenetic mechanisms that connect the molecular level to the phenotypic level through evolutionary and adaptive trajectories (Jorgensen 2011; Feinberg and Levchenko 2023; Hatakeyama and Ohbayashi 2024).

4) Connections with quantum phenomena.

Research into the intersection of ploidy variation, epigenetics, and quantum phenomena offers an innovative perspective on biological processes, particularly regarding cellular dynamics in highly sensitive, mutable, and adaptive systems. Since Löwdin’s (1963) pioneering proposal that proton tunnelling in DNA could have biological consequences, a substantial body of more recent work has indicated that quantum phenomena-such as tunnelling, coherence, and entanglement-play significant roles in biological processes including photosynthesis, DNA replication, and enzymatic catalysis (Marais et al. 2018; Kim et al. 2021; Slocombe et al. 2022). Understanding these phenomena, in conjunction with ploidy variation and epigenetic mechanisms, holds the potential to revolutionize our comprehension of the complexity and adaptability of living systems, while also opening new therapeutic avenues.

At the subatomic level, recent simulations by Slocombe et al. (2021) confirm that tunneling influences the stability of AT and GC base pairs. In alignment, Cooper (2009, 2011) suggests that enzymes such as transcriptase may “measure” quantum superposition states prior to decoherence. Additionally, studies in quantum biology show that organisms maintain quantum coherence in hydrophobic regions of proteins even under thermal stress (Hameroff and Tuszynski 2004; Vattay et al. 2014; Goushcha et al. 2014).

This biological coherence, as noted by Robert (2013), cannot be reduced to classical-quantum dichotomies and demands new models-as proposed by Bedau (2003) and Pezzulo and Levin (2016)-capable of explaining emergent properties that do not solely arise from local interactions. Moreover, mathematical models used to describe gene regulatory networks also exhibit parallels with quantum mechanics (Goh et al. 2020).

The concepts of entropy and the Gibbs-Boltzmann distribution, foundational in thermodynamics and statistics, have been used to model the quantum states of biological systems. In this context, the uncertainty and superposition observed in quantum systems can be seen as analogous to stochastic fluctuations and transitions between different attractor states in epigenetic landscapes (Jorgensen 2011; Asano et al. 2013). Thus, molecular fluctuations-or “biological noise”-may be interpreted in light of quantum principles, suggesting that seemingly chaotic biological phenomena can be analyzed through a quantum paradigm (Jorgensen 2011; Asano et al. 2013). This perspective reinforces the idea that biology operates through a complex interplay between classical and quantum dynamics (Jorgensen 2011; Asano et al. 2013; Kim et al. 2021; Goh et al. 2020; Bisiani et al. 2023).

The theory of quantum metabolism, which proposes the quantization of metabolic processes, also offers an analogy with atomic vibrations in crystalline solids-a concept traditionally addressed in physics (Davies 2006; Demetrius and Tuszynski 2010; Davies et al. 2012). The quantization of oscillatory modes in mitochondrial membranes, described by Davies et al. (2012), suggests that the process of ATP production follows patterns similar to those found in larger physical systems, such as atomic vibrations. This theory posits that metabolic rate and cellular energy efficiency can be described by laws analogous to those governing quantum systems, in which superposition states and decoherence are fundamental to understanding energy transduction processes.

This line of reasoning is increasingly framed under the umbrella of the “quantum mitochondrion,” a concept that brings together evidence for electron and proton tunneling through the respiratory complexes and long-range coherence within the inner mitochondrial membrane as contributors to the efficiency and regulation of oxidative phosphorylation, and that frames hormetic, stress-induced adjustments of mitochondrial function as shifts in this underlying quantum behavior (Nunn et al. 2016). Because mitochondrial function and ploidy-dependent metabolic reprogramming are tightly linked-as discussed above for hepatocytes and polyploid giant cancer cells-the quantum mitochondrion concept offers a direct mechanistic bridge between ploidy variation, epigenetic remodeling, and the quantum phenomena discussed in this section, rather than treating these as separate or merely analogous domains. Under this view, what is often described as mitochondrial “dysfunction” in polyploid or stressed cells may be more accurately understood as an adaptive shift in this quantum-mechanical operating regime in response to changing cellular demands, rather than as a simple breakdown.

The application of quantum principles to biology, such as proton tunneling, also provides critical insights into molecular stability and DNA replication (Slocombe et al. 2021; Casotti et al. 2024). Studies on DNA stability and quantum tunneling properties indicate that electron transfer and the maintenance of quantum coherence may influence the fidelity of genetic replication. This holistic view of biology, which considers the interaction between molecular fluctuations and quantum principles, has implications for evolution and cellular adaptation-especially in pathological contexts like cancer, where genomic and epigenetic alterations and molecular noise are intricately intertwined (Slocombe et al. 2021; Casotti et al. 2024).

**Fig. 1:**
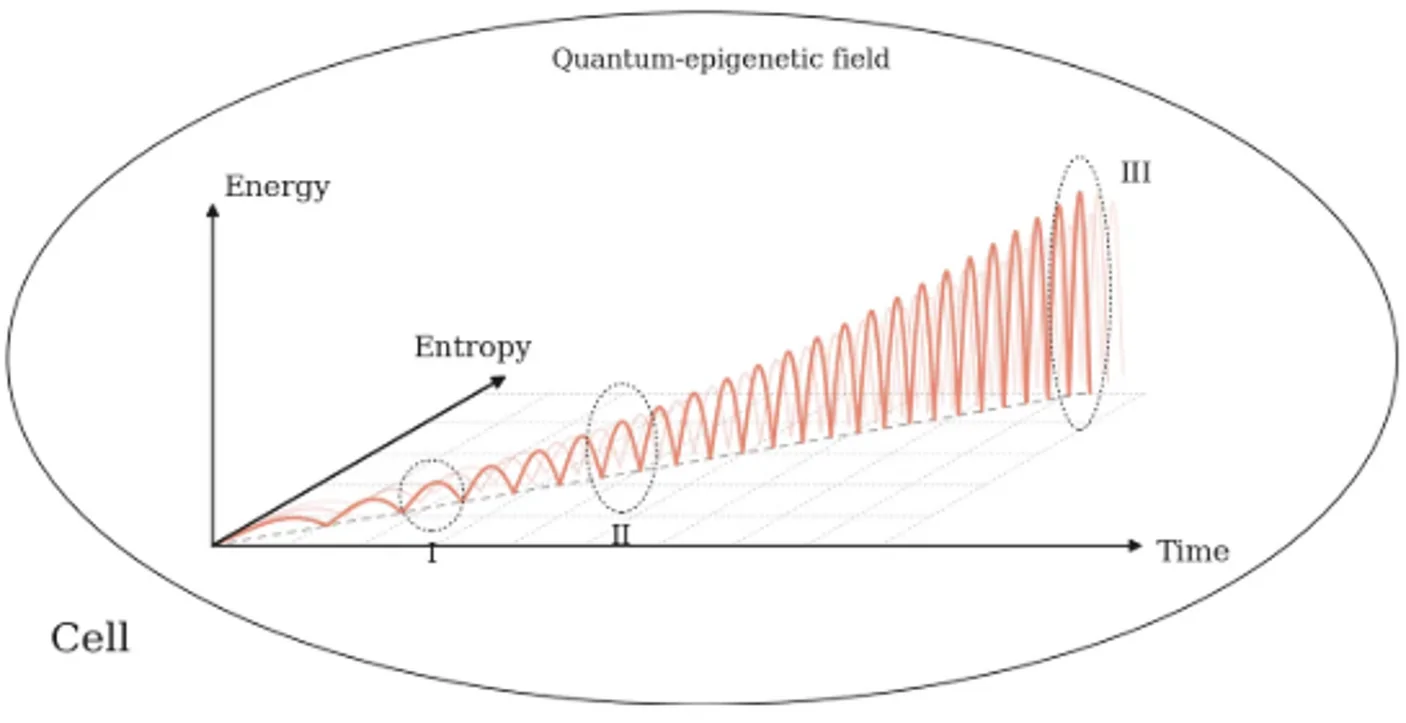
The self-amplifying oscillation: evolution of the cell within the quantum-epigenetic field across time, energy, and entropy

From a therapeutic standpoint, these discoveries open new possibilities for interventions targeting the regulation of metabolic and epigenetic states. Quantum modeling may be used to understand the dynamics of genetic and metabolic networks, enabling the development of more precise treatments for complex diseases (Caetano-Anollés et al., 2009; Demetrius et al. 2010). The synergy between ploidy variation, epigenetics, and quantum processes can, therefore, not only enhance our understanding of cellular adaptation mechanisms but also provide a robust theoretical foundation for advancing regenerative and personalized therapies (Caetano-Anollés et al., 2009; Demetrius et al. 2010; Boon et al. 2020; Casotti et al. 2024). In light of the aforementioned discussions, we propose that the interplay between ploidy-driven epigenetic landscapes and the underlying quantum-mechanical processes of the cell can be conceptualized as a Quantum-Epigenetic Field (QEF): a dynamic, multiscale framework in which quantum-mechanical principles-such as proton tunneling, coherence, and decoherence-constitute a foundational physical layer that, while general to matter as such, is considered here specifically as it operates within the biomolecular matter of living systems, while ploidy variation and epigenetic remodeling act as a higher-order, dissipative control system that modulates how these quantum processes are locally expressed within the cell. Rather than epigenetics generating the quantum environment, it is the genomic and epigenetic configuration of the cell that channels and constrains the quantum-mechanical “operating regime” available to its molecular machinery, thereby shaping the stability of attractor fields and the range of possible cellular outcomes, as illustrated in Fig. 1.

The Quantum-Epigenetic Field (QEF) Hypothesis and Biological Flexibility. The increase in genome size through polyploidy triggers a cascade of structural and functional reorganizations that reverberate throughout the entire cellular architecture, enhancing adaptive responsiveness and expanding the repertoire of gene expression through nonlinear interactions between redundant loci and epigenetic networks operating under regimes of quantum noise and thermodynamic stochasticity. In this case, what emerges is the presence of attractor fields of various distinct types (I, II, and/or III), configured within a landscape of dynamic emergent complexity resulting from polyploidization. However, a type I attractor field demonstrates a lower energy level with reduced entropy, essentially reproducing a normal biological system without significant influential noise. Nevertheless, under certain stress conditions, such as over time (the aging process), a process of senescence (interlinked with polyploidy) may occur, giving rise to type II attractor fields characterized by intermediate energy fluxes and an increase in entropy, which the system must compensate for. As temporal evolution proceeds and the system becomes increasingly dissipative, there is a tendency toward chaos, accompanied by an increase in energy flux and the emergence of attractor fields with quantum imbalances. Yet life simultaneously counterbalances this trend through the maintenance of new organizational orders, with adaptive landscapes shaped by random fluctuations and informational constraints that reorganize into more robust states. Thus, it becomes apparent that although life is a dynamic system in a transitional phase-where deterministic chaos, functional entropy, and quantum states coexist-polyploidy acts as a trigger for evolutionary bifurcations, opening new degrees of ontogenetic and phylogenetic freedom. There is a self-organization of living systems into highly functional metastable states, suggesting that life thrives in critical zones of controlled instability, where the collapse of quantum symmetries generates new forms of order and meaning. This process, governed by probabilistic laws and entangled retrocausal dynamics, points to a profoundly interconnected reality in which chaos, far from being mere noise to be eliminated, serves as the very foundation of biological diversity and creativity. Among everyday examples, it is worth highlighting adaptive randomness in immune, behavioral, and metabolic responses, as evidenced in protein synthesis, which alternates between deterministic models and random fluctuations generated by cell cycle events or the chemistry of enzymatic reactions. Here, the randomness parameter reflects dynamic disorder associated with enzymatic cooperativity, provoking paradoxical behaviors in which inhibitors may act as activators depending on substrate concentration and the presence of multiple rate-limiting steps within the reaction. In summary, multiple levels of control and nonlinear interactions point to a quantum-like mechanics of cellular decision-making, involving probabilities and superposed states that collapse into specific, often unpredictable, phenotypic responses. It should be clarified that the “time” axis of this schematic represents a multiscale continuum rather than a single timescale: at shorter, within-lifetime scales, it depicts developmental and physiological dynamics, such as the progressive shift from a low-noise type I attractor toward a type II attractor during aging and senescence; at longer, multigenerational scales, it extends across reproductive cycles, over which ploidy-driven reorganizations (type III) may become heritable and contribute to evolutionary diversification, thereby linking ontogenetic and phylogenetic levels of analysis. Created with PowerPoint and Canva.

5) Implications for extreme environments and astrobiology.

Based on insights into the definitions of life, the biological flexibility conferred by epigenetics and ploidy variation, as well as transformations associated with quantum alterations, the concept of life comes to be understood as a set of emergent properties. This view allows for extrapolating the traditional boundaries of identifying and projecting life into extreme environments-and even beyond Earth. A relevant aspect of this approach is the role of cellular plasticity in adaptation. Rather than considering life as a fixed entity, the idea of a dynamic and “reconfigurable” system is proposed, as previously discussed and further reinforced here, allowing organisms to flexibly adapt to planetary environmental conditions. This includes the concept of mutable organs or biological systems capable of responding to variations in available resources, extreme temperatures, and intense radiation-recurring characteristics of exoplanets (Hegner 2019; Helman 2022).

The astrochemical record reinforces this view of life as a phenomenon canalized by, rather than confined to, local conditions. Over the past decades, an increasing diversity of prebiotic organic molecules, including amino acid precursors and complete families of carboxylic acids involved in core metabolic pathways, has been detected or experimentally shown to form under the cold, low-density conditions of interstellar clouds and protoplanetary disks, and has even been inferred from the spectra of some of the most distant galaxies observed to date (Quilici et al. 2026). This suggests that the chemical “raw materials” for life are not a terrestrial peculiarity but are distributed throughout the universe wherever carbon, hydrogen, oxygen, and nitrogen chemistry can occur. Among these molecules, water occupies a singular position: beyond its role as a solvent, its hydrogen-bonded network exhibits quantum-mechanical behaviors-such as proton tunneling and nuclear quantum effects-that are increasingly recognized as intimately connected to the structure and function of proteins, nucleic acids, and membranes (Quilici et al. 2026). Because water with these properties is itself ubiquitous in the cosmos, the quantum-epigenetic considerations developed in this review need not be regarded as exclusive to Earth-based biochemistry, but rather as a plausible feature of any sufficiently water-rich, carbon-based chemical system.

Among several examples, the study of cryptobiosis stands out, as observed in organisms like tardigrades, showcasing a remarkable capacity for resistance to adversity. These organisms can survive extreme conditions-including extremely low temperatures, radiation, and even the vacuum of space-by entering a state of reduced metabolism (Des Marais et al. 2008; Hegner 2019; Helman 2022). This process of cryptobiosis illustrates a unique evolutionary adaptation, enabling life to persist even under conditions approaching total biological disintegration (Neuman 2006; Hegner 2019). This phenomenon inspires the development of biotechnological systems, such as the “Brownian ratchet,” which demonstrate how life can generate order from chaos, opening new avenues for biotechnology (Woese 2004).

By expanding the understanding of biology under extreme conditions, recent advancements have enabled the creation of artificial ecosystems capable of sustaining life in space environments. The application of concepts such as autopoiesis, as described by Yolles and Frieden (2021), highlights the ability of living systems to self-organize and maintain metabolic balance, even in states far from thermodynamic equilibrium. This property is essential for the colonization of exoplanets, such as Mars, where extreme conditions require adapted organisms capable of promoting terraforming, generating oxygen, nurturing ecosystems, and synthesizing materials from local resources (Igamberdiev and Gordon 2023; Ellis 2018).

At the same time, it is important to recognize that life as currently constituted remains canalized to the specific physicochemical conditions of its planet of origin. Even relatively short periods in low Earth orbit are sufficient to induce many of the molecular and physiological hallmarks of accelerated aging-including epigenetic age acceleration, altered telomere dynamics, mitochondrial stress responses, and changes in immune and DNA-damage profiles-driven primarily by microgravity, ionizing radiation, and the absence of a protective planetary magnetic field beyond low Earth orbit (Garrett-Bakelman et al. 2019). From the perspective developed in this review, this can be understood as a rapid loss of the ploidy-, epigenetic-, and quantum-level “calibration” that living systems have developed under terrestrial gravity, geomagnetic shielding, and radiation flux. Consequently, the colonization of other planets or moons is unlikely to be achieved simply by exposing terrestrial organisms to alien conditions and expecting indefinite adaptation; rather, as the autopoietic and dissipative principles discussed above suggest, sustainable colonization will likely require recreating, at least partially, the dissipative and energetic conditions to which terrestrial life is adapted, while simultaneously leveraging the kinds of ploidy-driven and epigenetic flexibility discussed throughout this review to extend the range of conditions under which life can persist.

The role of enzymes is equally fundamental in this adaptive process. As exemplified by Molina-Espeja et al. (2016), enzymes play a vital role in adapting biological systems to extreme environments. They are essential for the synthesis of DNA, RNA, and proteins, and for performing complex chemical transformations-even under conditions where other molecules fail. Combined with directed evolution, enzymes can be engineered to function in “alien” conditions, solving complex biological challenges and expanding the viability of life on exoplanets.

This is because enzymes catalyze reactions that remodel covalent bonds and emerge as dissipative metabolic networks exhibiting biomolecular oscillations, birhythmicity, and spatial waves (De la Fuente et al. 2021). These multi-enzymatic complexes, thermodynamically organized as open modules, process molecular information in real time via transfer entropy and form microcompartments that facilitate supramolecular self-assembly dependent on enzymatic kinetics (De la Fuente et al. 2021). Protein conformational fluctuations, modulated by hydration and the environment, enable dynamic conformations that can sustain coherent and entangled quantum states-as seen in hydrophobic pockets of actin and microtubules (Hameroff and Tuszynski 2004).

Moreover, proteins and enzymes operate in nonlinear, chaotic, and stochastic regimes, in which inhibitors can activate reactions under intrinsic noise (Ilan 2020). Variability in protein expression and copy number arises from intrinsic and extrinsic noise, including asymmetries in cell division, influencing cellular plasticity (Ilan 2020). For example, in cancer, energy shifts from structural construction to maximum dissipation in fractal entropy networks (Garland 2013), and quantum fluctuations can induce non-complementary malignant phenotypes, mediated by mutations that alter protein-DNA interactions and fractal sheets of entropic conductivity (Waliszewski 2022). Thus, enzymes and proteins are active agents of cellular self-organization, participating in pattern construction, energy regulation, functional coherence maintenance, and adaptive evolution.

Given these highlights on enzymes, biotechnology approaches based on genetic manipulation and enzymatic modulation may be promising areas of study for understanding and utilizing such agents as inducers of astrobiological adaptations. However, despite their promise, biotechnological advances raise important ethical and ecological concerns. Lombardo (2005) noted that genetic manipulation may lead to unpredictable impacts on ecosystems and involved organisms, reinforcing the need for careful and responsible approaches. Emerging technologies such as artificial cells and brain-computer interfaces challenge the boundaries between biological and artificial life, suggesting that these distinctions may become increasingly blurred in the future.

Furthermore, understanding modern cellular processes requires an integrated approach that simultaneously considers ploidy variation, epigenetic regulation, and quantum principles. Ploidy alterations, involving the number of chromosomal sets, generate structural diversity in organisms, creating a substrate for the action of epigenetic mechanisms. These mechanisms, as proposed by Jorgensen (2011), can be dynamically modulated, enabling flexibility in cellular behavior-similar to the idea of a “field of possibilities” in quantum systems. This suggests that biological adaptation in extreme environments may involve a complex interaction between these mechanisms, offering a new perspective on how organisms could evolve to survive in alien conditions.

These advancements underscore the importance of creating artificial biological systems. The exploration of exoplanets and space colonization depends on multidisciplinary biotechnological innovations to create biological systems adaptable to alien conditions. As illustrated by Rothschild et al. (2024), artificial biological systems can offer practical solutions for in situ resource production, which is essential for exoplanet colonization. Related work on lifelike computing systems explores how the self-organizing and self-repairing properties of organisms can be transferred to engineered infrastructures required to operate autonomously under such conditions (Stein et al. 2021).

The origin of life on Earth and its diversification may have been influenced by accidental chemical processes. However, the systemic foundations integrating chemistry, physics, and biology may hold the key to understanding how life could arise on other planets. Symbiogenesis, which involves the interaction of different organisms forming a superorganism, can be seen as a strategy for rapid adaptation to environmental changes, as discussed by Guerrero et al. (2013). Examples such as fungi and lichens, which show high resilience to extreme environments, provide clues about how organisms might evolve to survive on planets with conditions analogous to those found on exoplanets, a perspective in which even viruses can be read as agents of large-scale evolutionary and ecological change rather than as mere parasites (Gorbushina 2003; Sancho et al. 2008; Coleine et al. 2022; Simões et al. 2023; Grinin 2025).

Finally, the creation of artificial cells represents one of the greatest challenges in contemporary synthetic biology. Although significant progress has been made-such as the construction of multicompartment vesicles, minimal cytoskeletal systems, and artificial mitochondria-significant obstacles remain, particularly concerning membrane stability and self-sufficient replication (Göpfrich et al. 2018; Rothschild et al. 2024). In this context, Casotti et al. (2024, 2025) point out that tumor cells, due to their remarkable ability to adapt to dynamic environments, present a genetic instability that favors both resistance and survival-a feature that, although pathological, may offer valuable insights for developing more resilient and adaptive artificial biological systems.

6) Life in the quantum era: an emerging and transitional system.

This review aimed to integrate the vastness and complexity of the concept of life and to explore how it might be artificially designed, proposing a holistic vision that transcends traditional reductionist models. Life emerges as a dynamic and multifaceted phenomenon, situated at the intersection of biology, physics, and chemistry. In this context, life can no longer be viewed merely as a set of biochemical reactions but rather as a self-organizing system that evolves through complex interactions among its components-as suggested by Kauffman (1995), Corning (2022), and Bich (2020, 2021). Quantum physics, through the studies of Abbott et al. (2008), Hameroff and Tuszynski (2004), among others, expands the understanding that biological processes are deeply influenced by quantum phenomena-from genetic coding to cellular dynamics (Cooper 2009, 2011; Flavin et al. 2023). These approaches open new perspectives for molecular biology, especially when considering the interaction between epigenetics and quantum states, as described by Jorgensen (2011) and Asano et al. (2013), who demonstrate how cellular decisions are shaped by environmental contexts and quantum information.

Theories on the origin of life also align with this integrated perspective, highlighting that life is not a singular, isolated event but rather the result of a transition within an open physical system that, over time, generated complexity through quantum fluctuations and minimal energy states (Blackmond 2010; Goldenfeld and Woese 2011). The bottom-up construction of synthetic cells and the philosophy of artificial life expand the understanding of life as a system in constant evolution and adaptation, as exemplified in the works of Göpfrich et al. (2018), Helman (2022), and Dorin and Stepney (2024). Astrobiology, in turn, challenges the Earth-centric conception of life by proposing that life may emerge in extreme and non-terrestrial environments, with emphasis on the resilience of extremophile microorganisms (Gorbushina 2003; Des Marais et al. 2008; Hegner 2019).

This integration of approaches is reflected in the advancement of computational models, such as AlphaFold (Jumper et al. 2021), which have contributed to mapping the complex molecular interactions within cells, and in the growing interconnection between artificial intelligence and structural biology (Lane 2024; Korbak 2023). The concept of life, therefore, ceases to be something purely material or mechanical, becoming understood instead as an informational, energetic, and adaptive system-as advocated by Cruz-Rosas & Miramontes (2021), Luo and Zhang (2021), and Igamberdiev and Gordon (2023). Quantum biology, explored by Marais et al. (2018), reveals that phenomena such as photosynthesis and animal navigation cannot be adequately explained by classical physics, broadening the horizons for a deeper understanding of biological processes.

The philosophical view of life, as presented by Olby (1971) and Sims (2022), proposes that biological intelligence is oriented toward preserving organizational integrity-reinforcing the need for an approach that goes beyond reductionism. Pushing this line further, Minati (2023) asks in what sense properties such as intelligence and life may be understood as acquired systemic properties of matter itself rather than as attributes of particular substrates. This new paradigm, shaped by the convergence of biology, physics, epigenetics, and philosophy, not only challenges prevailing scientific paradigms but also offers new paths for understanding and treating diseases like cancer-whose origin can be reinterpreted as a collapse in the organism’s self-organization mechanisms (Rahman et al. 2014; Casotti et al. 2024, 2025). The integration of these diverse perspectives highlights the urgency of a transdisciplinary approach to the study of life-capable of exploring the dynamic interactions between bottom-up and top-down causalities, as suggested by Ellis, Noble, and O’Connor (2012).

Contemporary biology, therefore, demands a deeply integrated understanding of biological systems, moving beyond the traditional fragmentation of scientific disciplines. The proposal of a new ontology of life-in which self-organization, dissipative systems, and symbiogenesis are operational foundations-reflects the need to see life as an emergent phenomenon, organized by coherent and multiscalar interactions among its components. This concept redefines not only biology but also the way we understand the potential of biotechnology and synthetic biology. Complexity science has itself been analysed as a distinct scientific platform, with its own methods and institutional history, which helps to clarify what such an integrative programme can and cannot deliver (Vigni 2021); its explanatory reach extends to planetary-scale phenomena, as illustrated by systems readings of the interaction between oceans, ecosystems, and human health (Maumus 2020), with life understood as an emergent quality that can be modeled-and even created-in artificial contexts (Dodig-Crnkovic 2024; Dorin and Stepney 2024). This approach not only expands our understanding of natural life but also offers new possibilities for creating robust and innovative systems.

Two further points merit explicit discussion within this integrative framework. First, ageing itself can be understood not as an isolated pathological process but as an emergent property of life as a dissipative, self-organizing system operating within a particular environment: natural selection optimizes organisms for reproduction and population-level persistence rather than indefinite individual maintenance, so that the same dissipative dynamics that generate complexity and adaptability over evolutionary time also entail a gradual loss, at the level of the individual organism, of the regulatory “calibration”, spanning ploidy, epigenetic, bioelectric, and quantum-level parameters, discussed throughout this review. Viewed this way, ageing, regeneration, and cancer can be seen as different trajectories through the same underlying landscape of attractor states introduced in Fig. 1, rather than as unrelated phenomena. This framing suggests a natural extension of the model to age-associated diseases more generally. Within the landscape introduced in Fig. 1, conditions such as cancer and dementia may be represented not merely as a loss of function but as alternative attractors that become progressively accessible as the landscape is deformed with age. In the case of cancer, the reactivation of ancient unicellular and polyploidy-tolerant programmes described above (Davies and Lineweaver 2011) corresponds to a basin whose depth increases as genomic, epigenetic, and bioenergetic constraints relax, so that the malignant state becomes easier to enter and harder to leave. In neurodegeneration, by contrast, the relevant attractor appears to be a low-plasticity, high-maintenance-cost state in which post-mitotic cells can no longer sustain the metabolic and proteostatic work required to hold their configuration, and the system settles into a degraded but self-stabilizing regime. Framed in this way, ageing, cancer, and dementia become distinct trajectories over a single landscape rather than separate pathologies, and the framework yields a testable expectation: interventions that act on the parameters governing the shape of the landscape-ploidy distribution, methylation state, mitochondrial function, and bioelectric gradients-should alter the relative accessibility of these attractors, whereas interventions acting only on downstream markers should not. Such state-transition accounts of chronic disease are consistent with broader treatments of health and disease as dynamic, complex-adaptive states (Dinicola et al. 2011; Sturmberg 2021). Second, the boundary between “natural” and “artificial” life, repeatedly invoked throughout this review in the context of synthetic biology and astrobiology, may be less a binary distinction than a continuum defined by the degree to which a system exhibits the dissipative, self-organizing, and informationally adaptive properties discussed above; under frameworks such as assembly theory and autopoiesis, a system either exhibits these properties to a meaningful degree or it does not, regardless of whether its origin is described as natural or engineered; the same continuum is being explored from the side of design, where “livingness” is treated as a material quality that engineered artefacts may possess to varying degrees (Karana et al. 2020). Finally, this perspective invites a reframing of phenomena often described in purely negative terms, such as mitochondrial “dysfunction” in ageing or polyploid cells: rather than representing simple breakdown, these changes may often be better understood as adaptive reconfigurations of mitochondrial quantum-mechanical and metabolic operating regimes in response to the cell’s current ploidy, epigenetic state, and environment (Nunn et al. 2016).

**Fig. 2:**
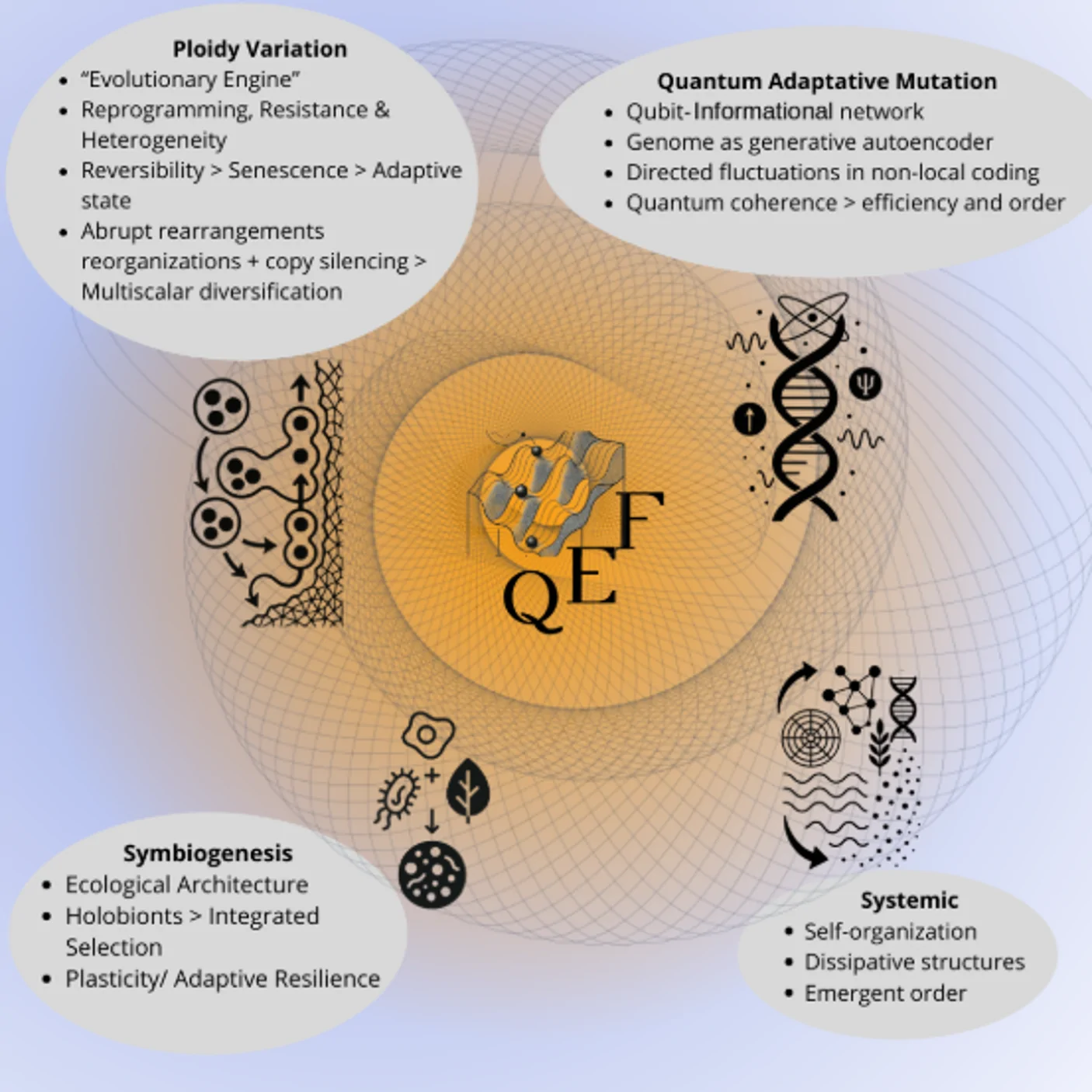
The Quantum-Epigenetic Field (QEF) as an integrating core: four convergent pathways between order and disorder

In short, the interconnection among biology, physics, chemistry, mathematics, and philosophy forms the foundation for a new epistemology of life-as advocated by Kim et al. (2021) and Flavin et al. (2023). This “era of quantum life” inaugurates a new phase of science, in which understanding life means intervening in living systems based on the foundational principles of these disciplines, with essential support from biotechnology and computing. Thus, it can be observed that the previous proposal regarding the presence and importance of a QEF may be interconnected with the emerging “era of quantum life.” To that end, we present a final compilation of all the ideas discussed throughout this review, as shown in Fig. 2.

Life in the quantum era through the QEF. Throughout the process of establishing life as we know it, four conceptual pillars stand out: (1) Systems biology, (2) Symbiosis, (3) Ploidy Variation, and (4) Quantum Theory. Ploidy variation (or polyploidy or whole-genome duplication) emerges as a central evolutionary phenomenon by imposing molecular and physiological adjustments, driving extensive chromosomal rearrangements, selective silencing, non-random retention or loss of redundant copies, reprogramming of gene expression, and acting as a driving force for diversification, a buffer against genomic perturbations, a catalyst for accelerated sequence evolution, and a source of functional specialization of gene categories. Accordingly, symbiotic relationships throughout the history of biological life potentiate the outcomes of polyploidy toward holobiont-level evolution, with integrated units evolving under natural selection, illustrated by vertical transmission and coevolution, further shaping phenotypic diversification. Thus, a connection arises between microevolution and macroevolution in the context of biological flexibility. In parallel, the impact of polyploidy on diversification provides a likely foundation for the redefinition of cell fate, especially when linked to dynamic epigenetic landscapes shaped by stochastic fluctuations and quantum phase transitions that integrate somatic mutations, tissue organization, and regulatory circuits into complex attractors reflecting quantum coherence and critical chaos. In this informational ecosystem, giant polyploid cells may serve as nuclei of extreme plasticity, where chaotic gene expression and bioelectromagnetic turbulence influence decisions between apoptosis, senescence or reprogramming, while mitochondrial dysfunction and quantum metabolism maximize entropy under adverse conditions, rendering cell fate reversible through dedifferentiation, transdifferentiation, and interventions sensitive to ion channels and long-range potentials, revealing a multiscalar morphogenetic organization that depends on non-trivial quantum effects and proton tunneling. Simultaneously, by viewing genomes as structures inspired by variational autoencoders that encode latent variables linking genotype and phenotype with adaptive fidelity, discontinuous C-value distributions and quantum patterns reflect evolutionary operators that resonate with epigenetic landscapes prone to identity collapse, anticipated by early warning signals mediated by SAM and acetyl-CoA. In this scheme, the genome is described as a generative autoencoder not because genomic information processing is itself a quantum computation, but because the encoder–decoder logic of autoencoders (compressing high-dimensional information into a lower-dimensional latent space and then reconstructing it) offers a useful analogy for how ploidy-driven epigenetic landscapes compress genotypic redundancy into a smaller set of regulatory “latent variables” that are subsequently “decoded” into phenotypes. Its placement within the Quantum Adaptive Mutation quadrant reflects the proposal that the directed fluctuations and non-local coding events that reshape this latent space are themselves influenced by the quantum-mechanical processes discussed throughout this review, rather than an assertion that autoencoders are quantum algorithms per se. This composes a dissipative self-regulating system in which methylation thermodynamics, critical decoherence, and structured quantum noise form an informational framework that governs the adaptive complexity and diversity of life. Created with PowerPoint and Canva.

## Conclusion

This review proposed an integrative and transdisciplinary approach to understanding life as an emergent phenomenon, organized through multiscale interactions among physical, chemical, and biological components. Concretely, this integration is exemplified by the Quantum-Epigenetic Field (QEF) framework proposed herein, which links a physical layer (quantum-mechanical processes such as tunneling, coherence, and decoherence), a chemical layer (epigenetic modifications and metabolic reprogramming), and a biological layer (ploidy variation, cell fate decisions, and organismal adaptation) into a single explanatory structure. Rather than merely juxtaposing findings from each discipline, this framework generates expectations that would not emerge from any single discipline in isolation-for instance, that changes in genome copy number should be accompanied by measurable shifts in the quantum-mechanical “operating regime” of key biomolecules, with downstream consequences for cell fate. By connecting ploidy variation, epigenetic regulation, and quantum phenomena, the study demonstrated that life can no longer be explained solely through reductionist models but must instead be viewed as an informational, adaptive, and self-organizing system. Ploidy, once considered a marginal aspect, has emerged as a central element in modulating cell fate and genomic plasticity. Its effects on genome stability, regenerative capacity, and metabolic adaptation highlight its importance in both evolutionary and pathological processes. When combined with epigenetic mechanisms-such as DNA methylation and histone modifications-ploidy promotes the emergence of diverse and functionally relevant cellular states. Moreover, principles of quantum physics-such as proton tunneling, molecular coherence, and state superposition-contribute to a deeper understanding of cellular dynamics, pushing the boundaries of classical biology. These insights are particularly relevant in contexts such as the origin of life, cancer, and the engineering of synthetic living systems. Extending this perspective to extreme and non-terrestrial environments, as explored in astrobiology, the article underscores the importance of life as a flexible and reconfigurable system. The creation of artificial life forms, inspired by natural principles, requires not only new technologies but also a paradigm shift in how we conceive what it means to be “alive”. Thus, this review suggests that the convergence of biology, physics, and philosophy may be the key to designing new forms of life and to deeply understanding living systems in all their complexity and transformative potential.

## Data Availability

No datasets were generated or analysed during the current study.
